# An Unusual Case of Compartment Syndrome of the Upper Extremity Caused by an iPad

**DOI:** 10.7759/cureus.14457

**Published:** 2021-04-13

**Authors:** Abhilasha Jyala, Niel Shah, Nisha Ali

**Affiliations:** 1 Internal Medicine, BronxCare Health System, Bronx, USA

**Keywords:** compartment syndrome, ipad, acute renal failure, acute renal failure, rhabdomyolysis and compartment syndrome

## Abstract

Acute compartment syndrome develops when intracompartmental pressure increases either due to intrinsic or extrinsic causes. Increase in compartment pressure eventually can lead to impaired tissue perfusion followed by tissue death if no urgent intervention is performed. Patients with acute compartment syndrome usually present with pain out of proportion to apparent injury. It can cause rhabdomyolysis, myoglobinuria, and eventually acute renal failure. The definite treatment is fasciotomy in a timely manner. We here report a very interesting case of acute compartment syndrome in a young patient cause by pressure over his axilla by an iPad.

## Introduction

Acute compartment syndrome develops usually after significant trauma, mainly after long bone fractures [1-3]. However, it may also develop following minor trauma or from nontraumatic causes. Any condition that can lead to a decrease in the volume capacity of a compartment or an increase in the volume of fluid within a compartment causes elevated intracompartmental pressure and thus makes a patient prone to compartment syndrome [4]. Usually, acute compartment syndrome is seen in younger patients under 35 years of age and more common in men than women [1]. The sex-specific difference in incidence can be explained by the relatively larger muscle mass of men contained within fascial compartments that do not change in size once growth is complete. Interestingly, patients who do not have any fracture but developed acute compartment syndrome due to other reasons are at higher risk for delayed diagnosis and thus delayed treatment (for example, fasciotomy) [5]. Here we present an interesting and unusual case of acute compartment syndrome in an intoxicated patient due to pressure over his axilla by an iPad.

## Case presentation

A 37-year-old Hispanic male patient presented to our emergency department (ED) with left chest wall and left shoulder swelling. This swelling was associated with left upper extremity weakness and left wrist drop. The patient reported that he was visiting his friend from Colorado and spent the night at a friend’s place drinking alcohol. He started feeling dizzy, went home, and fell asleep while using iPad in his bed. He slept on his left side of the body for the whole night and all day the day after. When he woke up, he found an iPad under his left axilla lying vertically and a bruise in his left axilla. He noticed swelling over his left side of the chest, left side of the lower face, and left shoulder. He was having difficulty in moving his left upper extremity with left wrist drop. On review of the system, the patient reported decreased urination and dark-colored urine. The patient did not have any medical comorbidity or history of any previous major surgery. He was not on any home medications and denied any known allergies. He reported drinking alcohol and smoking cigarettes socially but denied use of any illicit drugs.

The patient’s temperature, blood pressure, heart rate, respiratory rate, and oxygen saturation were within normal limits. Physical examination was significant for marked swelling of the left-sided chest wall, left shoulder, and left side of the lower face. The range of movement of the left upper extremity was extremely limited with left wrist drop. There were no sensory disturbances in the affected area, and left upper extremity pulses were intact. A bruise was noticed under the left axilla, and the patient had signs of dehydration. Physical examination otherwise was unremarkable.

Complete blood count was suggestive of leukocytosis (18,300 cells/uL) with neutrophilia, hemoconcentration (hematocrit of 52.7%), and normal platelet count (210,000/uL). Basic metabolic panel revealed hyperkalemia (potassium of 5.7 mEq/L), low serum bicarbonate (22 mEq/L), high creatinine (3.9 mg/dL), and high anion gap (19 mmoles/L). Liver function test showed elevated aspartate transaminase (AST) of 630 unit/L and elevated alanine aminotransferase (ALT) of 341 unit/L. Serum creatinine kinase (CK) was extremely elevated to 73,243 unit/L. Blood gas revealed respiratory and metabolic acidosis with pH of 7.23, and lactic acid was 3.9 mmoles/L.

**Figure 1:**
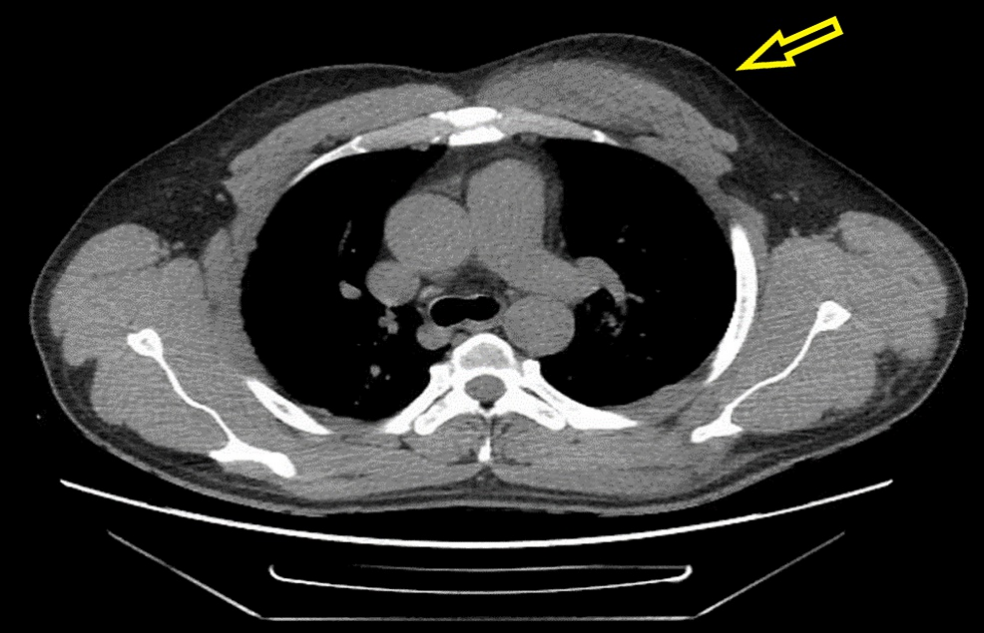
CT scan of the chest showing diffuse edema (yellow arrows) within the left chest wall compartment.

**Figure 2:**
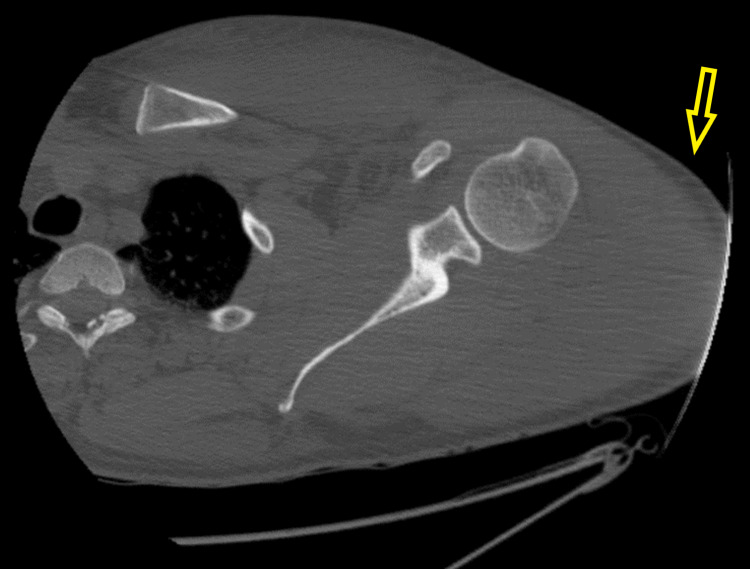
CT scan of the left upper extremity showing diffuse edema (yellow arrow) within the left shoulder compartment.

The patient was started on sodium bicarbonate drip and aggressive hydration for acidosis, rhabdomyolysis, and acute kidney injury (AKI) as per nephrology recommendations. The patient underwent CT scan of the cervical spine, chest, and left shoulder, which showed intramuscular hematoma involving the left pectoralis musculature and infiltrative changes involving the visualized left brachial plexus, without definite large soft tissue hematoma (Figures 1, 2). The patient was admitted to the intensive care unit for further management of rhabdomyolysis, AKI, severe acidosis, suspected brachial plexus injury (likely Saturday night palsy), and possible compartment syndrome. He was regularly followed by nephrology, orthopedics, and vascular surgery services during the hospital course. The patient was managed conservatively with hot/cold compressions over the affected part, but later he underwent anterior left arm compartment and left shoulder compartment fasciotomy due to failure of conservative management. Intercompartmental pressure measurements were 15 mmHg in the anterior deltoid, 19 mmHg in the middle deltoid, and 14 mmHg in the posterior deltoid. Meanwhile, rhabdomyolysis was improving but AKI was getting worse, and thus the patient was started on intermittent hemodialysis by nephrology. MRI of the brachial plexus was performed and ruled out any brachial plexus injury. The patient continued to be on intravenous fluids and showed improvement in his renal function. He showed mild improvement in left extremity motor function, and fasciotomy wound was closed after 10 days. The patient was advised to follow up with physical therapy and occupational therapy as an outpatient. 

## Discussion

Compartment syndrome usually develops secondary to any condition that increases the volume of the compartment without an increase in the diameter of the unyielding myofascial envelope. The rise in interstitial tissue pressures can be due to either intrinsic factors (e.g. swelling or bleeding) or extrinsic factors that restrict the ability of the fascial envelope to expand, or both [6]. Acute compartment syndrome of the lower extremity is more common than of upper extremity. The most affected sites in the lower extremity and the upper extremity are calf and forearm, respectively. The need for fasciotomies is less if the upper extremity is involved (approximately only 20% of all extremity fasciotomies) compared to when the lower extremity is involved [7,8].

The common causes of compartment syndrome, which can involve both upper and lower extremities, include long bone fracture, acute extremity ischemia with reperfusion, crush injury, burn injury, hematoma formation, soft tissue infection, systemic inflammatory response syndrome, massive fluid resuscitation, nontraumatic myositis, and rhabdomyolysis. Other causes that are more common for upper extremity compartment syndrome include high-pressure injection, birth injury (neonatal compartment syndrome), intravenous extravasation injury, and inadvertent intraarterial injection. On the other side, causes such as prolonged immobilization and snakebite can more commonly involve the lower extremity [8]. However, immobilization was the cause for upper extremity compartment syndrome in our case. The causes of compartment syndrome can also be classified into intrinsic and extrinsic causes [6]. Intrinsic causes, such as intercompartmental bleeding or edema, can increase the volume within the compartment and thus can raise compartment pressure. Extrinsic causes include tightly applied casts, tight dressings, anti-shock garments, closure of fascial defect, intraoperative lithotomy positioning, and lateral positioning. In such cases, removal of an extrinsic factor, such as casts or circumferential dressings, or repositioning can improve symptoms and may obviate the need for fasciotomy. In our case, in addition to immobilization and intoxication, extrinsic factor (iPad) also played an important role in causing acute compartment syndrome of the upper extremity.

Acute compartment syndrome in extremities can also develop in unconscious or obtunded patients with prolonged limb compression from soft tissue injury and swelling [9,10]. Most commonly it occurs in the lower extremity, but cases involving the upper extremity have been reported as well. The two most common examples in which prolonged immobilization causes acute compartment syndrome are lithotomy positioning during surgery and obtundation after an intoxication, which was seen in our case. Additionally, gluteal compartment syndrome has also been reported after prolonged immobilization in morbidly obese patients undergoing bariatric surgery and intoxicated individuals who are immobilized for a prolonged period [11].

**Figure 3:**
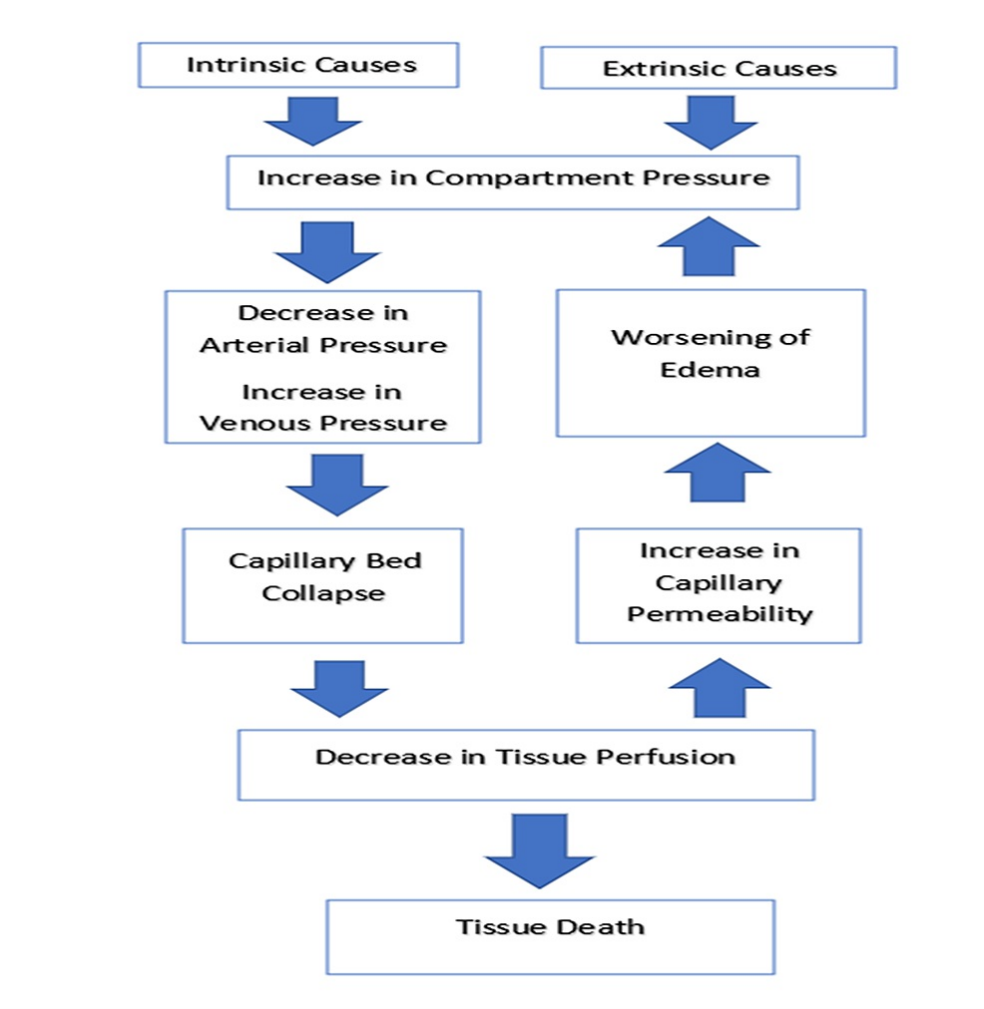
Pathophysiology of acute compartment syndrome. The figure has been modified for this publication. Original figure is from: Oak NR, Abrams RA. Compartment syndrome of the hand. Orthop Clin North Am 2016; 47:609. Illustration used with the permission of Elsevier Inc. All rights reserved.

When a limb is positioned against an immobile object, it may decrease local tissue perfusion and predispose to the development of acute compartment syndrome [12]. The other factors associated with an increased risk of developing positioning-related acute compartment syndrome include prolonged immobility, systemic hypotension, and morbid obesity [13,14]. Each of these variables decreases local muscle perfusion, creating the potential for ischemia-reperfusion injury. The pathophysiology of acute compartment syndrome is summarized in Figure 3.

Common clinical features of acute compartment syndrome include pain out of proportion to apparent injury (early and common finding), persistent deep ache or burning pain, and paresthesia (onset within approximately 30 minutes to two hours). The findings suggestive of acute compartment syndrome include tense compartment with firm (wood-like) feeling, pain with passive stretch of muscles in the affected compartment (early finding), decreased sensations, pallor, muscle weakness, and paralysis (late finding). If left untreated, it can lead to sensory deficits, muscle contractures, paralysis, infection, fracture nonunion, and possible need for limb amputation. Additionally, patients with acute compartment syndrome can have rhabdomyolysis, which leads to myoglobinuria and eventually acute renal failure, which may require dialysis.

Acute compartment syndrome is mainly a clinical diagnosis that can be complemented by measurement of compartment pressures. Usually, laboratory values have no value in making the diagnosis. If the diagnosis is suspected, urgent surgical consultation and the measurement of compartment pressures should not be delayed obtaining a laboratory result. As described earlier, laboratory abnormalities may include elevated CK levels secondary to rhabdomyolysis, which can lead to myoglobinuria and eventually can cause elevated creatinine level.

The most important aspect of diagnosis is to keep a high index of suspicion among patients at risk for acute compartment syndrome and frequent serial examinations. The surgical team should be consulted immediately when there is a suspicion of acute compartment syndrome, and when it is not possible (remote areas and hospitals with limited surgical coverage), the patient should be transferred immediately to another hospital where compartment pressures can be measured and fasciotomies performed. The complications associated with acute compartment syndrome can be avoided by early diagnosis and appropriate treatment.

The immediate management of suspected acute compartment syndrome mainly includes relieving all external pressure on the compartment such as any dressing, splint, cast, or other restrictive covering should be removed. The affected limb should be kept at the heart level to avoid arterial inflow and dependent swelling, which can exacerbate limb ischemia [15]. The patient should receive analgesics and supplemental oxygen. Also, hypotension should be avoided in such patients by treating it with boluses of intravenous isotonic saline as hypotension decreases perfusion and exacerbates tissue injury.

The definitive treatment of acute compartment syndrome in most cases is fasciotomy to decompress involved compartments. The morbidity can increase with delays in performing fasciotomy, including the need for amputation [16]. In some cases, such as in cases where the muscle is already dead, fasciotomy will not provide and benefit but will increase the risk of infection. Definitive treatment for such injuries often involves amputation [17]. Additionally, hyperbaric oxygen has been described as adjunct treatment for acute compartment syndrome [9,18]. However, further studies are needed to determine the appropriate role of hyperbaric oxygen therapy.

## Conclusions

We can conclude that acute compartment syndrome is commonly seen in young males and usually develops most commonly after a trauma. However, it is not unusual to develop acute compartment syndrome in the absence of trauma as in our case, which involves prolonged immobilization in an intoxicated patient along with continuous pressure over his axilla by an extrinsic factor such as iPad. The common clinical features of acute compartment syndrome include pain out of proportion to injury, paresthesia, and pallor, which can even lead to paralysis. Other associated features include rhabdomyolysis, myoglobinuria, and acute renal failure. Urgent surgical evaluation for the need for fasciotomy is the most appropriate management of acute compartment syndrome. Hyperbaric oxygen has been described as a useful adjunct treatment, but this option needs to be evaluated in future studies.
